# Chitosan Hydrogel Supports Integrity of Ovarian Follicles during *In Vitro* Culture: A Preliminary of A Novel Biomaterial for Three Dimensional Culture of Ovarian Follicles 

**DOI:** 10.22074/cellj.2020.6393

**Published:** 2019-07-31

**Authors:** Fatemeh Hassani, Bita Ebrahimi, Ashraf Moini, Ali Ghiaseddin, Mahshid Bazrafkan, Gholamreza Hassanzadeh, Mojtaba Rezazadeh Valojerdi

**Affiliations:** 1 Department of Anatomy, School of Medicine, Tehran University of Medical Sciences, Tehran, Iran; 2 Department of Embryology, Reproductive Biomedicine Research Center, Royan Institute for Reproductive Biomedicine, ACECR, Tehran, Iran; 3 Department of Gynecology and Obstetrics, Roointan-Arash Maternity Hospital, Tehran University of Medical Sciences, Tehran, Iran; 4 Department of Endocrinology and Female Infertility, Reproductive Biomedicine Research Center, Royan Institute for Reproductive Biomedicine, ACECR, Tehran, Iran; 5 Biomedical Engineering Group, Faculty of Chemical Engineering, Tarbiat Modares University, Tehran, Iran; 6 Department of Anatomy, Faculty of Medical Science, Tarbiat Modares University, Tehran, Iran

**Keywords:** Alginate, Chitosan, Hydrogel, Ovarian Follicle

## Abstract

**Objective:**

Testing novel biomaterials for the three dimensional (3D) culture of ovarian follicles may ultimately lead to a culture model which can support the integrity of follicles during *in vitro* culture (IVC). The present study reports the first application of a chitosan (CS) hydrogel in culturing mouse preantral follicles.

**Materials and Methods:**

In this interventional experiment study, CS hydrogels with the concentrations of 0.5, 1, and 1.5% were first tested for fourier transform infrared spectroscopy (FT-IR), Compressive Strength, viscosity, degradation, swelling ratio, 3-(4,5-dimethylthiazol-2-yl)-2,5-diphenyltetrazolium bromide (MTT) cytotoxicity and live/dead assay. Thereafter, mouse ovarian follicles were encapsulated in optimum concentration of CS (1%) and compared with those in alginate hydrogel. The follicular morphology, quality of matured oocyte and steroid secretion in both CS and alginate were assessed by enzyme-linked immunosorbent assay (ELISA). The expression of folliculogenesis, endocrine, and apoptotic related genes was also evaluated by quantitative real-time polymerase chain reaction (qRT-PCR) and compared with day that in 0.

**Results:**

The rates of survival, and diameter of the follicles, secretion of estradiol, normal appearance of meiotic spindle and chromosome alignment were all higher in CS group compared with those in alginate group (P≤0.05). The expression of *Cyp19a1 *and *Lhcgr* in CS group was significantly higher than that of the alginate group (P≤0.05).

**Conclusion:**

The results showed that CS is a permissive hydrogel and has a beneficial effect on encapsulation of ovarian follicle and its further development during 3D culture.

## Introduction

More patients have survived thanks to the developments in cancer treatment; however, chemotherapy and/or radiation, for instance, can produce acute or chronic ovarian insufficiency. Nonetheless, fertility in at risk for premature ovarian failure (POF) and young women can be preserved in a number of ways (1). Recording over 70 live births, ovarian tissue transplantation has shown to be a successful procedure for fertility restoration among adults (2). Notwithstanding, for cases which incur a risk of reimplantation of malignant cells, cryopreserved ovarian tissue transplantation is not a safe method. Individual follicle culture (IVC) can be a good alternative for transplantation, which can be cultured through twodimensional (2D) or three-dimensional (3D) systems. Eppig and Schroeder’s pioneering work led to the first live mouse offspring from *in vitro* matured (IVM) follicles grown on collagen gels (3).

In the conventional 2D culture systems, ovarian stromal cells move from around the oocyte and spread on the culture vessel surface and thereby interfering with stromal cell-oocyte interaction (4). The chief impediment to the procedures of IVM in this system has been the ineffectiveness of oocyte development. Hardly 2D culture system can preserve the follicle 3D architecture and the complex interaction between the components of the somatic cell and the oocyte that is required for nuclear and cytoplasmic maturation. Also, a critical element for the transport of particular amino acids to the oocyte and sharing of paracrine factors is the gap junctions between the oocyte and its surrounding granulosa cells. Likewise, oocyte-derived secretions contributes to the regulation of metabolic processes and proliferation of granulosa cells (3). A great many recent studies have attempted to design 3D culture systems through application of hydrogel biomaterials which can grant biomechanical and biochemical support to ovarian follicle cultures (5). Some researchers have used various hydrogel materials, such as agarose (6), alginate (7) collagen (8) and hyaluronic acid (9) for 3D encapsulation of isolated follicles in different animal models. Alginate has proved the most appropriate biomaterial in various animal species for its positive results with follicles (10). Although there are some data affirming follicle culture in alginate and morphologically normal oocyte production, a new study has argued that the meiotic spindle assembly might suffer from disturbance due to the lower developmental competence of oocytes obtained from the alginate system (11). This fascinating finding give grounds for the time spent on the quest for a fundamentally different biomaterial for the 3D culture. Chitin, as a biomaterial commonly found in insect and crustacean exoskeletons and fungi cell walls, is the second most abundant natural polymer which can be used as a scaffold for culturing follicles. Chitin application is generally carried out using its deacetylated form, chitosan (CS), which is composed of glucosamine and N-acetyl glucosamine linked in a β manner (12-14). Crucial elements for describing the characteristics of CS are degree of deacetylation and molecular weight, which vary depending on source and process of production (13). CS has been widely used in biomedical applications within the two past decades for its suitability for cell in growth, biodegradability, osteoconduction, porous structure, intrinsic antibacterial nature, and biocompatibility (15). On that account, CS has been used extensively, for instance, in cartilage tissue engineering (16), wound healing (17), drug delivery system (18) and orthopedic applications (15). The present study reports the first application of a CS hydrogel in culturing mouse preantral follicles.

## Materials and Methods

In this interventional experiment study, the Ethical guidelines set by Royan Institute was considered during all stages of the experiment (IR.ACECR.ROYAN. REC.1395.197).

### Preparation of chitosan hydrogels

Three CS solutions (medium molecular weight, 75- 85% deacetylated, Sigma Aldrich, USA); 0.5, 1 and 1.5% (w/v) were prepared through dissolution of CS powder in acetic acid 1% (w/v) in phosphate-buffered saline (PBS, Gibco, USA) at room temperature and magnetic stirring for 5 hours at 150 rpm. The pH of solutions was adjusted to be around 6.8-6.9 by adding NaOH (10 N, Merck, Germany) to the stirring solution. To change the phase from solution to gel (pH=7.2-7.4), NaOH solution (0.075 N) was added at pH=6.8 after dripping of CS solutions in microtiter wells.

### Scanning electron microscopy

To investigate the pore structure of the CS gels, the samples were immersed (0.5, 1, and 1.5%) in 4% glutaraldehyde in PBS at 37˚C, removed after 1-day, washed with PBS and frozen at -80˚C followed by lyophilization (Zibrus technology GmbH, Germany) for 72 hours. Having gold nanoparticle sputtered on the samples’ surfaces and scanned at an accelerating voltage of 20 kV using scanning electron microscopy (SEM, Seron, Korea), pore diameter of the dried hydrogels was obtained via image analysis using Image J software (version 1.44 p national institute of health).

### Fourier transform infrared spectroscopy

The chemical bonds linking the CS and other chemicals were analyzed using Fourier transform infrared (FT-IR) spectroscopy analysis, an account of which was given in the hydrogel preparation process. In brief, the samples were dried in a vacuum oven for 72 hours (BINDER GmbH, Germany), grinded and mixed with potassium bromide for 1:100, then prepared a compressed disk with the thickness of 1mm. The FT-IR spectra in a wavelength range of 4000-400 cm^-1^ were acquired applying an FT-IR spectrometer (Thermo Nicolet, USA).

### Compressive strength of hydrogel

In order to determine the elastic modulus, a compression test was applied to the cylindrically-shaped samples. The hydrogel samples were prepared based on the aforementioned method and were then tested between two compression plates under a rate of 5 mm per minute at room temperature (n=3) in a compressibility study instrument (Santam, Iran). The stress-strain curve was drawn and Young’s modulus calculated to demonstrate the mechanical strength of the samples.

### Hydrogel degradation

The hydrogel degradation rates were measured by preparing 500 μl precursor CS solutions in 2 mL Eppendorf tubes. After the gelation process was complete, 1 ml of the DMEM medium was loaded in each tube. The tubes were incubated in a shaker incubator at 100 rpm and were subsequently sampled at 0, 0.25, 0.5, 1, 2, 4, 8, 16, 32, 64, 128, 256 hours (n=3 per time point). The medium was changed every 48 hours (1 ml was removed and then replaced with a fresh medium). One ml of the medium was taken from the tubes and the gels were then frozen at -80˚C. The samples were lyophilized along with each other for 72 hours and were weighed, and the weight loss rate was recorded.

### Swelling studies

Through immersing lyophilized CS samples in PBS at 37˚C, swelling rate of hydrogels was determined. The sample weights were recorded at particular intervals (n=3 per time point) until no weight change was observed. Afterwards, towel papers were used to blot the swollen samples and were immediately weighted. The swelling ratios (Q) were determined as a percentage of the swollen gel weight increment, with the dry polymer weight serving as the reference.

### Viscosity of hydrogel precursor solutions

CS samples in solution phase were transported and measured volumetrically. Workability of sol phase of samples was studied by measuring the viscosity of CS precursor solutions (0.5, 1, and 1.5%) at 30˚C (n=3) using viscometer (Brookfield, DV-III Ultra, USA).

### MTT cytotoxicity assay

The hydrogel samples from 100 μl of three different precursor solutions were prepared in each well of 96 well plates and an approximate number of 100000 human bone marrow mesenchymal stem cells (hBMMSCs), provided by Royan Institute, were seeded on the surface of each hydrogel sample. The samples were incubated in Dulbecco’s Modified Eagle’s medium (DMEM, Gibco, USA) and supplemented with 10% fetal bovine serum (FBS) under standard cell culture conditions in 96 microwell. Then hydrogel cytotoxicity and proliferation were investigated by MTT-assay (Gibco, USA) after 0 1, 3, 5, 10 days of incubation (n=3 per time point). The culture medium was changed every other day.

### Live/dead assay

The proliferation potential and cell viability were studied via live/dead assay by applying acridine orange (AO) staining (Sigma-Aldrich, Germany). Images were captured with inverted fluorescent microscope (Nikon, Japan). It is noteworthy that double stranded DNA with AO emits green light, whereas single stranded DNA reveals dead cells and RNA becomes orange to red with AO.

### Alginate hydrogel preparation

Sodium alginate (Sigma, USA) was dissolved in deionized water to a concentration of 1% (w/v). Then, to remove organic impurities and to improve the purity of alginate, it was purified with activated charcoal (0.5 g charcoal g alginate). After treatment with charcoal, the sterile alginate solution was filtered through 0.22 μM filters, then diluted with 1×PBS at the rate of 0.7% (4, 19).

### Animals and ovarian follicle isolation

Twelve-day-old female Naval Medical Research Institute (NMRI) mice were obtained from Royan Institute animal house. They were kept in a controlled temperature (20-to- 25˚C) and light-controlled environment (12 hour light: 12 hour dark). Mice were sacrificed by cervical dislocation; ovaries were isolated and early prenatal follicles were mechanically dissected from the ovaries utilizing 28 gauge needles. Isolated follicles were immersed in α-minimal essential medium (α-MEM, Gibco, USA) supplemented with FBS (5 mg/ ml) at 37˚C in 5% CO2 in air. Only follicles with 100-130 μm diameter, intact and visible immature centrally located oocyte round were selected for further experiment. Prior to encapsulation, individual follicles were maintained in α-MEM, 5% FBS at 37˚C, 5% CO_2_ for 45 minutes.

### Follicle encapsulation and culture in chitosan and alginate hydrogel

First, 5 μL beads of alginate were placed on a petri dish (Falcon, USA) and individual preantral follicles were inserted into the beads. After that calcium bath [140 mM NaCl (Sigma, USA) and 50 mM CaCl_2_ _2H_2_O (Sigma, USA)] was added to 5 μL beads of alginate solution containing preantral follicle for 3 minutes. For CS groups (0.5, 1, and 1.5%), NaOH solution (0.075 N) was added at pH=6.8. to change solution state to gel (pH=7.2-7.4). First, 5 μL beads of CS were placed on a petri dish (Falcon, USA), having already been neutralized with 0.5 μL of NaOH solution (0.075 N) and washed three times by PBS– and α-MEM. Then 3 μL of solution CS were placed on a petri dish; one follicle plunged into the bead; follicle was removed from CS solution by pipette pasture and then was put on in the center of neutralized CS bead that plunged in α-MEM (follicle embedding). CS solution was cross-linked with OH sodium bicarbonate in culture medium. Finally, each bead was cultured in 96 wells containing 100 μL of culture medium and was incubated at 37˚C and 5% CO2 for 13 days. Every 3-4 days, 40 μl of culture medium was exchanged with fresh medium.

### *In vitro* maturation of preantral follicles and ovulation induction

A total of 187 follicles were divided into, alginate (93 follicles, 6 replicates) and CS (94 follicles, 6 replicates) groups. Encapsulated follicles in 2 groups including alginate 0.7% and CS 1% were cultured individually in a 96-well plate (TPP, Switzerland) for 13 days. Culture medium was composed of α-MEM supplemented with 5% FBS, 5 mg/ml insulin, 5 mg/ml transferrin and 5 ng/ml sodium selenite (ITS, Gibco, UK), 10 mIU/ml recombinant-follicle stimulating hormone (r-FSH, Merk, Germany). An inverted microscope with transmitted light and phase objectives (Nikon TI) was utilized to assess follicle survival and diameter (ImageJ softwere). Those follicles which no longer had their oocytes surrounded by a granulosa cell layers or their granulosa cells had become dark and fragmented and their follicles had decreased in size were considered to be dead. Diameter of the follicles was assessed on days 6 and 13 of the culture. IVM oocytes were induced on day 13 of culture by adding 2.25 IU/ ml human chorionic gonadotropin (hCG). After 16-18 hours, maturation was assessed through an inverted microscope by presence of the first polar body. A treatment was carried out for denudation of the oocytes from the surrounding cumulus cells with 0.3% hyaluronidase (Sigma-Aldrich, St. Louis, MO) and gentle aspiration via a polished drawn glass pipette. The oocytes were checked to see whether they had germinal vesicle breakdown (GVBD) for the cases where germinal vesicle was not in sight. The oocytes were categorized as metaphase II (MII) if there was a polar body in the perivitelline space; shrunken or fragmented oocytes were categorized as degenerated (DG). In this phase, CS 1% was determined to be an optimum concentration. Thus, all assessments for CS 1% were compared with alginate 0.7%.

### Immunocytochemistry

MII oocytes from the *in vitro*-cultured follicles and also in vivo oocytes (from adult mice) as a control were obtained and fixed in 4% paraformaldehyde at room temperature for 1 hour, permeabilized in PBS with 0.1% Triton X-100 and 0.3% bovine serum albumin (BSA) for 15 minutes, and blocked in PBS comprising of 0.01% Tween 20 and 0.3% BSA. The oocytes were incubated in anti-tubulin for visualization of the spindle: fluorescein isothiocyanate (anti-tubulin: FITC; 1:150; Abcam, USA) for 1 hour, and cortical granules (CGs) were detected with rhodamineconjugated Lens culinaris agglutinin (1:200; Vector Laboratories, USA) during incubation with antitubulin: FITC. At the end, DNA was stained by 1 μg/ mL 4’,6-diamidino-2-phenylindole (DAPI, Sigma, St. Louis, MO, USA) for 5 minutes. Images were taken using a fluorescence microscope (Eclipse 50i, Nikon, Japan).

### Hormone assays

Androstenedione, 17β-estradiol and progesterone were measured in conditioned media collected on follicle culture (day 1 and 13) using commercially available radioimmunoassay kits (ELISA kit, BT Lab, China). The condition media obtained from each time point of the specific cultures were pooled together (15-20 samples pooled per measurement). The sensitivities for the androstenedione, estradiol and progesterone assays were 0.1 ng/ml, 10 ng/ml and 0.1 ng/ml, respectively.

### Gene expression and RNA extraction

To evaluate gene expression, follicles in the experimental and control groups were collected in four replicates: on day 0 (control group, 20-30 preantral follicles in each replicate), and day 13 (experimental groups, 15-20 antral follicles in each replicate) of culture, pooled in Cell Reagent RNA Protect (RNeasy kit, Qiagen, Germany) and stored at -70˚C until RNA extraction. Total RNA was extracted from each of the separated follicular pools using an RNeasy Micro Kit (Qiagen, Germany) according to the manufacturer’s instructions. cDNA was also synthesized using Revert Aid H Minus First Strand cDNA Synthesis Kit (Takara, Otsu, Shiga, Japan) and random hexamers according to the manufacturer’s instructions.

### Quantitative real-time polymerase chain reaction

mRNA was extracted and cDNA was synthesized, and was then amplified using master mix which was composed of specific oligonucleotides and TAKARA SYBR Green (Takara, Otsu, Shiga, Japan). Oligonucleotide primers used are listed in Table 1. Samples were analyzed for gene expression of folliculogenesis *Ggf9, Bmp15, Zp3, Cx37, Cx43, Bmp4* and *Bmp7*, endocrine genes *Lhcgr, Cyp11a1, Cyp17a1, Cyp19a1* and *Fshr*, as well as for apoptotic genes such as *Bax, Casp3, Bcl2,* and *P53* (Table 1). Gapdh was considered as the housekeeping gene. Data was representative of four independent experiments.

### Statistical analysis

The statistical analysis was carried out using SPSS version 16 (SPSS Inc., Chicago, IL, USA). All experiments were performed with three independent biological replicates, and the data, with the exception of meiotic competence and hormone assays, were analyzed using one-way ANOVA, followed by Tukey’s test. The meiotic competence and hormone assay data were analyzed using t test. Differences were considered significant at P<0.05.

## Results

### Scanning electron microscopy analysis

**Fig.1:**
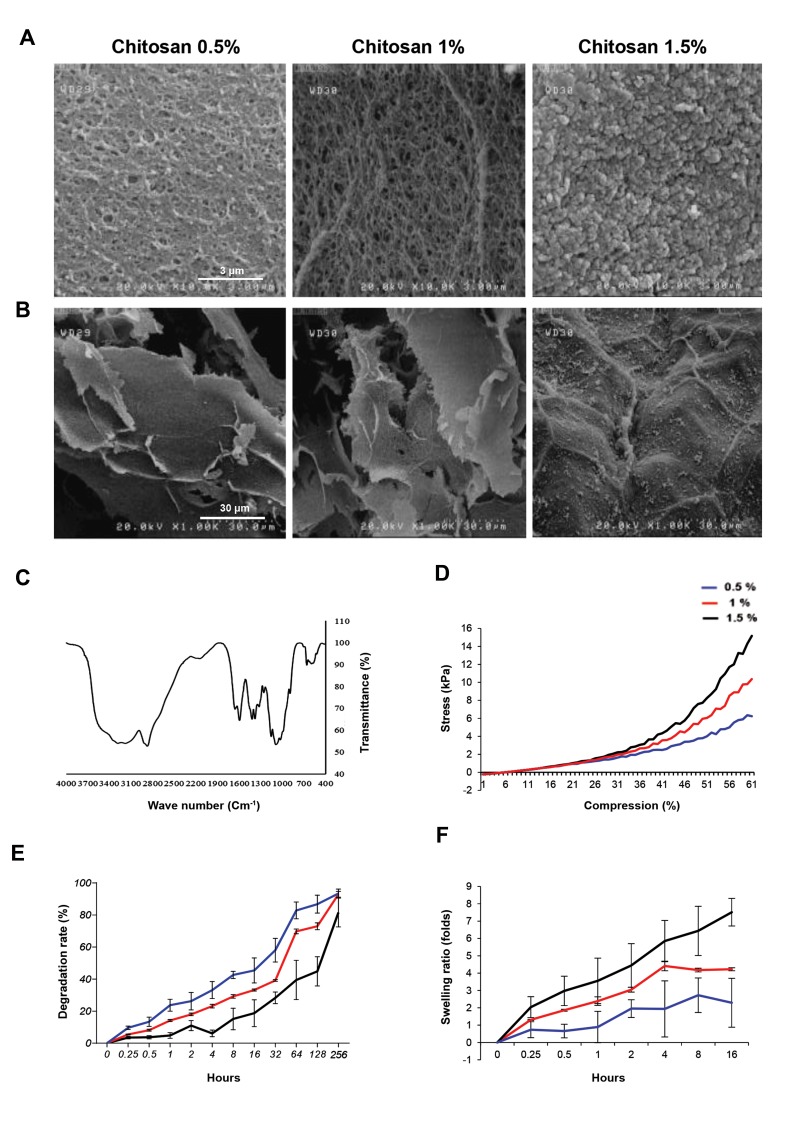
Mechanical and structural assessment of chitosan hydrogels. **A, B.** The scanning electron microscopy (SEM) images of morphology and porosity of chitosan hydrogels (scale bars A: 3 μm, B: 30 μm), **C.** FTIR spectra of chitosan hydrogels, **D.** Compressive strength of chitosan hydrogels, **E.** Degradation rate of the chitosan hydrogels, and F. Swelling ratio of the prepared chitosan hydrogels.

Figures 1A show that the 0.5% and 1% CS samples were porous with almost homogeneous porosity which made sheet format of solid fibers. The sheets distance is about 25 μm which creates enough room for cell nests. In higher concentration of CS 1.5%, in hydrogel, the fibril solid content is much higher which, as it can be seen, there is no free space between in the layers. Moreover, in high resolution images, Figure 1B, the nano-pores about 300 nm are visible at 1% and 0.5% of CS where, high concentration of CS at 1.5% prevents the formation of these pores.

### Fourier transform infrared spectroscopy analysis

As it was described in our previous article (20), FT-IR spectra in Figure 1C show stretching vibration of C—H at 663. There are peaks for hydroxy groups and N—H group starching. Peak bend on 517, 643, 851 and 1093 cm-1 are showing presence of PO4 3- which means successful gelation using ionic gelation process in the phosphate buffer. Peaks decreasing at ~1090 cm-1 and C—H bands with the centrality of ~2880 cm-1 reveal the presence of CH3COONa made of NaOH reaction with acetic acid.

### Mechanical strength and elastic modulus of hydrogels

The compressibility strength of the hydrogels in the three concentrations is shown in Figure 1D. The average peaks of stress for each concentration, 0.5, 1, 1.5% samples, at breaking point were 6.2, 10.3 and 15.5 kPa, respectively. The strain, length gradient over initial characteristics length, was 53.5, 51.4 and 52.6% for 0.5%, 1% and 1.5%; also, Young’s modulus of the three samples was 30.8, 19.8, and 10.3 kPa, respectively.

**Table 1:** Primer sequences used for real-time polymerase chain reaction analysis


Gene	Primer sequence (5´-3´)	Product length (bp)	Accession number

*Gdf9*	F: CAAACCCAGCAGAAGTCAC	194	NM_008110.2
	R: AAGAGGCAGAGTTGTTCAGAG		
*Bmp15*	F: AAATGGTGAGGCTGGTAA	148	NM_009757
	R: TGAAGTTGATGGCGGTAA		
*Zp3*	F: CTTGTGGATGGTCTATCTGAG	125	NM_011776.1
	R: GTGATGTAGAGCGTATTTCTG		
*Gja4 (Cx37)*	F: CGACGAGCAGTCGGATTT	155	NM_008120.3
	R: AGATGACATGGCCCAGGTAG		
*Gja1(Cx43)*	F: TAAGTGAAAGAGAGGTGCCCAGA	200	NM_010288.3
	R: GGTTGTTGAGTGTTACAGCGAAAAG		
*Bmp4*	F: GGTCGTTTTATTATGCCAAGTCC	417	NM_001316360.1
	R: ATGCTGCTGAGGTTGAAGAGG		
*Bmp7 *	F: CTATGCTGCCTACTACTGTGAG	103	NM_007557.3
	R: GTTGATGAAGTGAACCAGTGTC		
*Trp53 (p53)*	F: AACTTACCAGGGCAACTATG	203	NM_001127233.1
	R: TGTGCTGTGACTTCTTGTAG		
*Casp3 *	F: AAAGACCATACATGGGAGC	138	NM_001284409.1
	R: CGAGATGACATTCCAGTGCT		
*Bax *	F: TTGCTACAGGGTTTCATCCAG	246	NM_007527.3
	R: CCAGTTGAAGTTGCCATCAG		
*Bcl2*	F: GCCTTCTTTGAGTTCGGT	162	NM_009741.5
	R: ATATAGTTCCACAAAGGCATCC		
* Fshr*	F: ACGCCATTGAACTGAGATTTG	134	NM_013523.3
	R:GAACACATCTGCCTCTATTACC		
* Lhcgr*	F: AAGCACAGTTAGAGAAGCGA	244	NM_013582.3
	R: GGTCAGGAGAACAAAGAGGA		
* Cyp11a1*	F: TCCTTTGAGTCCATCAGCAG	180	NM_001346787.1
	R: GTCCTTCCAGGTCTTAGTTCT		
* Cyp17a1*	F: AGAAGTGCTCGTGAAGAAGG	201	NM_007809.3
	R: TTGGCTTCCTGACATATCATCT		
* Cyp19a1*	F: ATGTCGGTCACTCTGTACTTC	107	NM_001348171.1
	R: TTTATGTCTCTGTCACCCACAAC		
* Gapdh*	F: GACTTCAACAGCAACTCCCAC	125	NM_001289726.1
	R: TCCACCACCCTGTTGCTGTA		


### Hydrogel degradation

Figure 1E presents weight losses in the samples in DMEM over time. The three hydrogels could lose weight in the DMEM solution because of the solvent penetration and the growing swelling pressure on the mechanical strength arising from physical cross-linking points in the polymeric network. This resulted in a gradual decline in the structural integrity of the ionically and physically cross-linked hydrogels, leading to disintegration and collapse of the hydrogels in the media. In addition, due to modest and progressive drop of ambient pH to 6.5 and the hydrogels weight loss, there was a simultaneous transition of the gel-sol. Figure 1E shows the gradual weight loss of the samples in the early hours due to gel structure integrity. As could be seen in the figure, there was a steady increase in the gel-sol transition and rate disruption and after 20 days, with the 0.5% sample losing more than 90% of its weight. The two other samples reflected a similar case as well. The more weight loss of the 0.5% sample was affirmed by the mechanical strength test, samples morphology, and porosity.

### Swelling ratio

According to Figure 1F, swelling ratio for 0.5 and 1% samples was high; however, it gradually decreased. The maximum swelling for the two concentrations is about 10 hours, but in 1.5% hydrogel even after 16 hours there is an increasing trend in the volume of hydrogel which could be attributed to high mechanical strength of the higher concentrations. The equilibrium swelling ratios for the two concentrations were 360 and 415, respectively after around 10 hours.

### Viscosity of chitosan solutions

The viscosity of hydrogel precursor solutions was 12.5 and 20 cp for 0.5 and 1% samples, respectively. Whereas the parameter was not measurable for 1.5% sample due to its high viscosity which resembled a solid scaffold. The 1% sample had a higher viscosity for presence of more mineral salt in the precursor solution. On the whole, the results suggest that the viscosity of the 1% sample suited the in situ gel formation as follicle seeds during sol-gel transformation.

### Cytotoxicity of hydrogels

**Fig.2:**
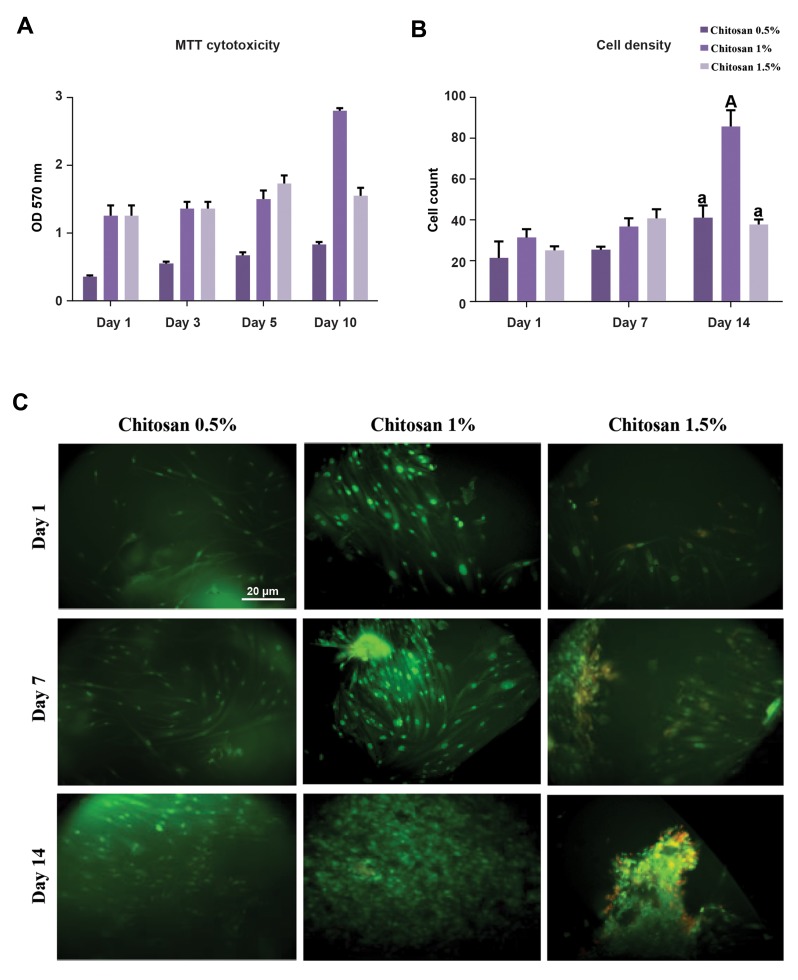
Hydrogel cytotoxicity and viability of hBMMCs were investigated by MTT-assay and acridine orange staining respectively. A. MTT-assay, B and C. Viability of cells was performed by live/dead staining acridine orange after 1, 7 and 14 days of cell culture (scale bar: 20 μm). The data were analyzed using ANOVA test. Capital letters versus same small letters (A with a) indicated significant difference (P<0.05). hBMMCs; human bone marrow mesenchymal stem cells and OD; Optical density.

Figure 2A shows the cytotoxic effect of hydrogels on hBMMSCs which were seeded on their surface via MTT-assay for 10 days. The survival of cells is reported in terms of optical density (OD) at 570 nm. These results show that the hydrogels were non-toxic for hBMMSCs, and biocompatible for 31, 45 and 36% cell growth during this time for the samples. The OD and proliferation of cells seeded on 1% sample were more than what were observed for the other samples. It could be explained by considering the fact that 1% sample contained culture medium more than 1.5% sample and it has more available surface for cell proliferation rather than 0.5% sample. With respect to time standard and given that 75% of the primary cells survived after the MTT test, it can be said that all the hydrogels were biocompatible and non-toxic.

### Cell viability

The viability rates for the hBMMSCs, which were cultured in the 1% sample on days 1, 7 and 14, were 90.76, 95.45, and 94.97, and in the 1.5% sample 87.5, 95.04, and 91.30, accordingly. There was no significant difference regarding the number of viable cells of the two samples; however, there was a significant difference between the samples for the dead cells (P<0.05) on the 14^th^ day of culturing (Fig .2B). Figure 2C presents the results for AO staining concerning hBMMSCs which was used to fill the hydrogel samples after 1, 7 and 14 days since cell culture was initiated. As the figures show, the majority of the cells were alive and fully dispersed and stretched in the hydrogels. There were remarkable survival rates for live/dead staining.

### Biocompatibility and testing of chitosan concentrations for follicle culture

**Fig.3:**
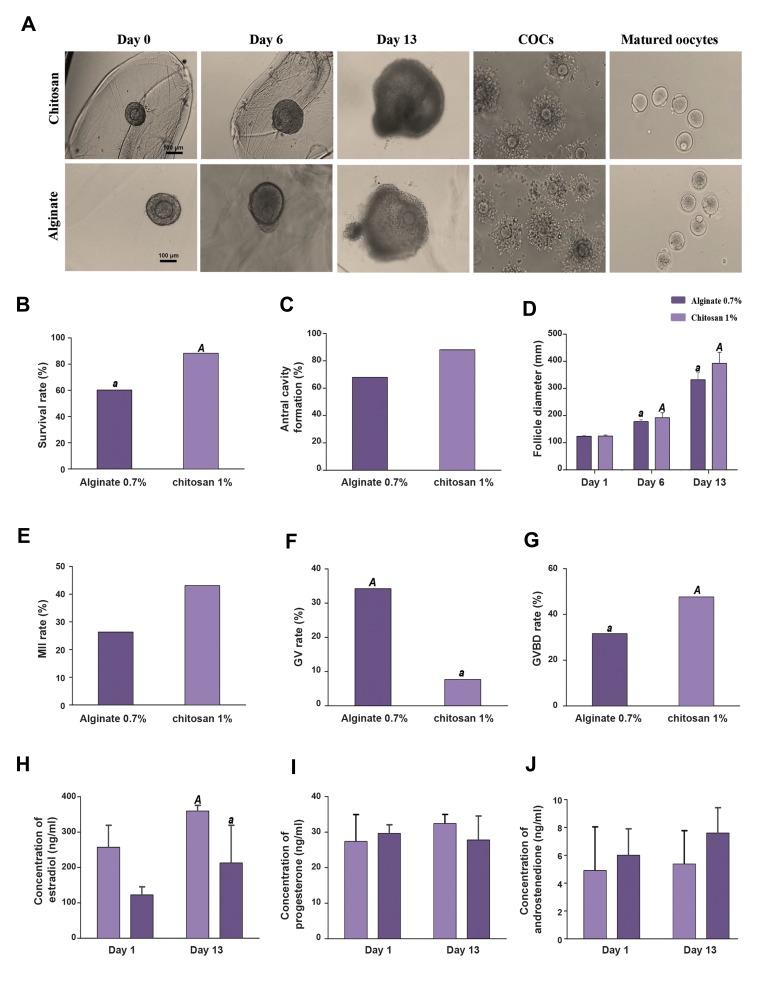
Morphology of follicles from day 1 to day 13, meiotic competence and hormone secretion. A. Morphology of preantral follicles was embedded in 1% chitosan (scale bar: 100 μm) and alginate hydrogel (scale bar: 100 μm) from day 0 to day 13. B. Survival rate, C. Antral formation rate, D. Follicle diameter, E. MII, F. GV, G. GVBD rate, H, I, and J. Estradiol, progesterone, and androstenedione secretion into the culture medium. As the graph depicts, estradiol and progesterone increased on day 13 in follicles from chitosan 1% when compared with alginate however, estradiol was significantly higher than alginate. The data were analyzed using t test. Capital letters versus same small letters (A with a) indicated significant difference (P<0.05). Day 0; An early secondary follicle, Day 6; Eccentric oocyte movement within the follicle, Day 13; Antral cavity formation is clearly visible, COC; Cumulus-oocyte complex, MII; Metaphase II, GV; Germinal vesicle, and GVBD; Germinal vesicle breakdown.

When NaOH was added to CS 0.5%, gel form did not compose that was because of the low viscosity. In addition, the severe stiffness of CS 1.5%, did not allow encapsulation. No obvious sign of cytotoxicity such as oocyte extrusion and cellular degeneration could be noticed after 24 hour of culturing. CS supported the *in vitro* follicular growth and granulosa cell proliferation for 13 days. Encapsulated follicles in 1% concentrations of CS continued to follicular diameter increase during the growth phase and they retained their spherical morphology (Fig .3A). As Figures 3B and 3C depict, survival rate was significantly lower in alginate compared to CS 1%. In addition, antral cavity formation was higher in CS 1% than alginate group but it was not significant.

### Characterization of follicle growth and oocyte meiotic competence in chitosan hydrogel

Typical morphology of preantral follicles and oocytes after 3D culture in CS hydrogel is shown in Figure 3A, CS embedded secondary follicles maintained their 3D spheroid architecture throughout IVC. An intact basal lamina with a thecal cell layer was typically observed (Fig .3A, day 13). The diameter of the follicles in CS was significantly higher than that in alginate by day 6 and day 13 (day 6: 192 ± 18.81 versus 177.5 ± 7.37 μm, day 13: 392.25 ± 40.7 versus 332.3 ± 26.3 μm, respectively) (Fig .3D).

After 13 days of culture, follicles were separated from alginate, CS groups, and induced with hCG for 16-18 hours. Oocyte quality produced from engineered follicles in the CS and, alginate hydrogels was measured by their ability to continue meiosis, as shown by GVBD and MII oocytes (Fig .3A). MII rate was higher in the CS 1% than the alginate group (43.08 versus 26.3%); however, no significant difference in the MII rate was found among the two groups (Fig .3E). Oocytes from alginate 0.7% showed significantly higher GV (34.2 versus 7.6%) and lower GVBD (31.57 versus 47.63) rate than the CS group (P<0.05) (Fig .3F, G).

### Steroid secretion

Ovarian hormones including estradiol, androstenedione, and progesterone were secreted by the theca and granulosa cells throughout the follicle development. At the end of the culture, follicles in CS 1% group secreted significantly more estradiol compared to alginate group (Fig .3H) (P<0.05), and Progesterone levels increased, but this increase was not significant (Fig .3I). In CS 1%, androstenedione level decreased on day 13 compared to alginate but was not significant (Fig .3J). In two groups all hormones increased non-significantly throughout the culture period and in the meantime of culture, progesterone decreased slightly in alginate group.

### Gene expression analysis

#### Folliculogenesis genes

**Fig.4:**
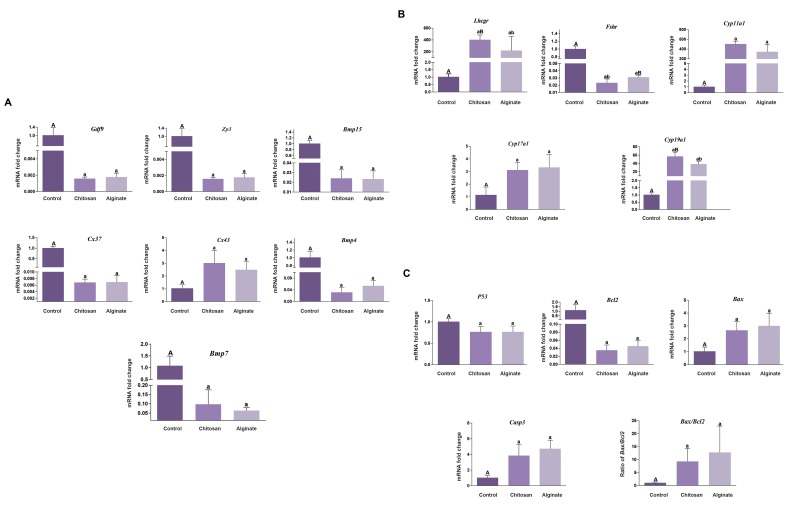
Gene expression analysis. A. Expression levels of folliculogenesis genes (*Gdf9, Zp3, Bmp4, 7, 15, Cx37* and *Cx43*), B. Expression levels of endocrine genes (*Lhcgr, Fshr, Cyp11a1, Cyp17a1, Cyp19a1*), and C. Expression levels of apoptotic genes (*P53, Bcl2, Bax,* and *Casp3*). GAPDH was regarded as the internal control. Data were analyzed using the one way ANOVA. Capital letters versus same small letters (A with a, B with b) indicated significant difference (P<0.05).

The expression of seven genes involved in folliculogenesis such as *Ggf9, Bmp15, Zp3, Cx3, Cx43, Bmp4* and *Bmp7* were assessed. In CS and alginate groups, these genes had similar patterns of expression relative to day 0 as a control group. In CS and alginate hydrogel, the expression of all folliculogenesis genes, with the exception of *Cx43*, decreased significantly from preantral to antral stage with a fold change of at least 16 (P<0.05, Fig .4A). *Cx43* had 2-3 fold increased expression in CS and alginate groups, relative to the control one (P<0.05).

#### Endocrine-related genes

The expression of five genes involved in endocrine such as mRNA encoding aromatase Cyp19a1, Cyp11a1, Cyp17a1, the FSH receptor (Fshr), Lhcgr was assessed. In CS and alginate groups, five endocrine genes had similar patterns of expression relative to day0 as a control group. In CS and alginate hydrogel, all endocrine-related genes expression, with the exception of Fshr, increased from preantral to antral stage with a fold change of at least 3 (P<0.05, Fig .4B). However, *Lhcgr, Cyp11a1,* and *Cyp19* expression levels during *in vitro* culture (IVC) in CS were higher than alginate. The expression of *Cyp19a1* (1.51 fold) and *Lhcgr* (1.82 fold) had significantly increased in the CS group relative to alginate. *Fshr* had 0.5 fold decreased expression in CS which was significant compared to alginate (P<0.05).

#### Apoptotic-related genes

The expression of four genes involved in apoptosis such as mRNA encoding *Bax, Bcl2, Casp3, P53,* and ratio of *Bax/Bcl2* was assessed. In CS and alginate groups, these genes had similar patterns of expression relative to day 0 as a control group. In CS and alginate hydrogel, the expression of *P53, Bcl2,* decreased significantly from preantral to antral stage with a fold change of at least 1.5 (P<0.05, Fig .4C). *Bax* and *Casp3* and Bax/ Bcl2 had 3 fold increased significantly expression in CS and alginate groups, relative to control group (P<0.05).

### Spindle and chromosome alignment, cortical granule formation

**Fig.5:**
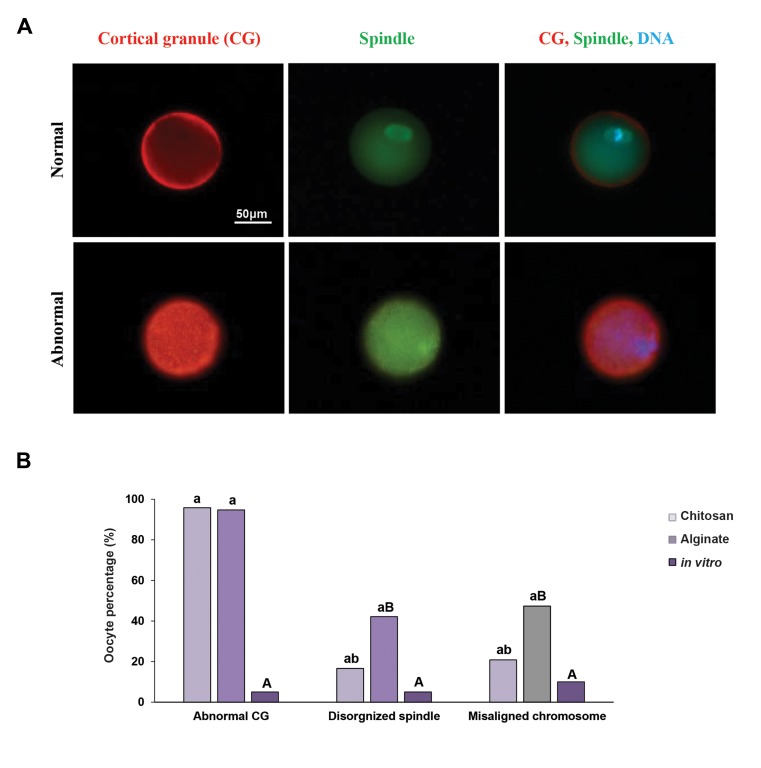
Spindle organization, chromosome alignment and cortical granule (CG) formation. A. MII oocytes were obtained from the ovaries of NMRI mice following culturing in the alginate and chitosan hydrogels. They were fixed and stained for DNA (DAPI, blue), tubulin (green) and analyzed for chromosome alignment and spindle organization. These oocytes were compared with oocytes which developed in vivo and were then matured in vitro (scale bar: 50 μm) and B. The experiment was performed three times, and at least 20 oocytes were analyzed for each group. Data were analyzed using ANOVA test. Capital letters versus same small letters (A with a, B with b) indicated significant difference (P<0.05).

The weakened developmental competence of the in vitro follicle culture derived oocytes, could pose problems in chromosome alignment on the spindle and spindle formation (Fig .5A). Oocytes from alginate hydrogel group showed 42.1% of spindle disorganization and 47.36% of them had at least one chromosome located outside of the metaphase plate, which was significantly higher than in vivo MII oocyte as a control group and CS hydrogel, in which only 10% of spindle disorganization and 5% of chromosomes misalignment were seen in control group (P<0.05). In CS hydrogel, 16.6% of MII oocytes had a disorganized spindle and 20.83% had at least one chromosome located outside of the metaphase plate, which was not significantly higher than those of control group (Fig .5B). Cortical granules, the secretory granules found in CG exocytosis and oocytes play a role in inducing zona pellucida blocking to avert polyspermy (21) . While CGs in the control group display a uniform cortical distribution in the MII oocytes (Fig .5A), CGs present in MII oocytes derived from in vitro cultured follicles in alginate and CS hydrogel were clumped and did not display a uniform cortical distribution (94.73 and 95.8%, respectively) and this was significantly higher than control group (5%) (P<0.05, Fig .5A, B).

## Discussion

This study aimed to elucidate the first application of a CS hydrogel to the 3D culture of ovarian follicles. To obtain optimal concentration of CS, 0.5, 1, and 1.5% (W/V) were used for ovarian follicular culture. FTIR tests confirmed the chemical stability of this type of in situ gel formation according to a study conducted by (20). It is supposed that the microstructure and swelling properties of scaffolds have significant effect on cell penetration, proliferation, and function in tissue engineering (22). The average pore size of scaffolds decreased by the increase in the hydrogel concentration. CS 1% showed homogeneously distributed pores and higher degradation ratio compared to CS 1.5%. On the whole, the swelling ratio of scaffold could be attributed both to the microstructure and hydrophilic nature of scaffold (23). The high hydrophilicity of CS may also have contributed to the high degradation of this hydrogel. Scaffold also was needed to have sufficient rigidity to maintain the 3D structure of the follicle and to coordinate follicle development. Antrum formation and steroidogenesis are two aspects of this developmental process which are affected by the scaffold (4). The elasticity of the CS hydrogels was altered by making adjustments in the concentration of the solution. The results of our study revealed that CS 1% was an optimum concentration for ovarian follicular culture. The higher swelling ratio of 0.5% sample and SEM images compared with that of 1% sample can be attributed to its weak mechanical strength.

The investigations demonstrated that varying the matrix physical properties would change the microenvironment from permissive to nonpermissive for follicular culture. The mechanical properties of the matrix and the hydrogel elasticity are two crucial elements that can directly influence the phenotype and function of the in vitrocultured ovarian follicles (24-26). In this study, the elastic modulus for CS 1% was 19.8 kPa. M. Xu et al. (24), suggested that Matrices with a shear modulus of less than 250 Pa are considered as permissive since a large percentage of follicles cultured in these matrices survive and increase in diameter; develop an antrum; have a steroidogenic profile similar to that of follicles in vivo, and produce MII stage oocytes. On the other hand, matrices with a shear modulus greater than 500 Pa are considered as nonpermissive since they decrease the rate of antrum growth and formation, as well as alter steroid production, thereby decreasing the oocyte quality. Based on these studies and results obtained in our study, CS is a permissive hydrogel because its shear modulus is less than 250 Pa and lesser than alginate 0.7% (203 pa). Antral cavity formation, survival and MII rate, diameter of follicles on day 6 and day 13 are higher in CS 1% than in alginate 0.7%.

In hormone production, 17β-estradiol hormone is produced by the mature ovarian follicle and maintains oocytes in the ovary (24, 27). At the end of the culture, follicles from CS1% secreted more estradiol compared with those in alginate. Our data showed that immature ovarian follicles progress to mature form and secrete 17β-estradiol hormone. The expression of genes associated with steroidogenesis and antrum formation was also explored to find out their patterns as a function of the matrix properties.

Time and matrix conditions were regarded as two factors affecting the variation in the detected levels (26). Thus, the expression of the following genes related to steroidogenesis during day 0 and day 13 of follicle culture and culture in two different matrix conditions, alginate 0.7% and CS 1%, was examined. In both groups, the folliculogenesis, endocrine, and apoptotic genes, had similar patterns of expression relative to the control group. *Cyp19a1* and *Lhcgr* had increased expression levels within the CS follicles compared with alginate. During development, *in vivo*, follicles demonstrated increasing *Cyp19a1*, which is responsible for the conversion of androstenedione to estradiol expression as they mature and approach ovulation (28, 29). As the mature follicles approach ovulation, there is expression of in vivo, *Lhcgr* in the mature follicles. The follicle is capable to respond to the LH surge for the upregulation of LHCGR, which results in ovulation, granulosa cell luteinization, and cumulus mucification (30). Consequently, expression of *Lhcgr* throughout the IVC is an indication of follicle maturity. The upregulation of *Lhcgr* in the permissive follicle culture took place on Day 6 of culture, which was in harmony with the timing of antrum development and follicle differentiation. In contrast to *Lhcgr, Fshr* was expressed in both mature and immature granulosa cells (31), with a stabilized expression level during the follicle development at the mRNA and protein levels (32, 33). In the same line with these in vivo observations, the *Fshr* expression did not undergo any alterations throughout the culturing, but in this study *Fshr* expression was downregulated in CS and alginate groups relative to control group. Also this gene had 0.5 fold reduced expression in CS compared with alginate. Thus, it can be concluded that in the last stage of follicular growth (preovulatory), the follicle would grow to its maximum and do not require *Fshr* gene expression, but on the other hand, for ovulation, the gene expression of *Lhcgr* should be high. *Gdf9* and *Bmp15* (Tgfb super family) are two oocyte-secreted proteins which are present in all follicular stages (34). Expression levels of the aforementioned genes decreased during 12 days of culture (35). Also in this study expression patterns of *Gdf9* and *Bmp15* were similar to those found in previous study (36). *ZP3* and *Bmp4*, 7 were essential in the follicular development and had an expression pattern similar to that in *Bmp15* and *Gdf9* in this study (37). This transcript decline could be translated into the rise in the protein synthesis at the end of the follicle culture, which could be regarded as a proof for a better growth of the follicles in the 3D culture system (36).

Forces were generated by follicles in 3D matrices as a result of outward force exerted by the growing follicle on its surrounding matrix, which was reciprocated by the surrounding matrix at a magnitude depending on the stiffness of the matrix and the force exerted by the follicle. The resulting increased pressure on the follicle may account for the apoptosis and/or the decreased proliferation rates of follicle cells (38).

Apoptosis is a dynamic process which might occur during IVC of follicles Apoptosis, a kind of programmed cell death, has been associated with a range of processes which deal with normal functions of the follicular and ovary development, including atresia and corpus luteum regression (39). Ratio of *Bax/Bcl2* in alginate group was higher than that in CS group, but this difference was not significant. This result demonstrated that stiffness of CS 1% was more suitable for 3D culture of preantral follicle to antral than alginate 0.7% hydrogel.

According to the results obtained in this study, CS system supports nuclear maturation, alignment of chromosomes, and organization of spindle; however, alginate system could not support them. Both of the systems, alginate and CS, could not support the cytoplasmic maturation. In the control group, CGs displayed a uniform cortical distribution, but they were clumped in CS and alginate groups. Thus, CG biogenesis/localization appeared to be impaired in these oocytes. The results of this study also confirmed the findings reported regarding the fact that in alginate hydrogel there might be some disturbance in meiotic spindle assembly and cortical granules (11).

The primary reason accounting for the low efficacy of IVM in assisted reproduction would be inadequacies of the culture media used. The evidence shows nuclear maturation in human oocytes is properly supported by the culture systems, but these systems cannot produce oocytes with cytoplasmic maturation, consequently, reducing the potential for embryo development (40). Therefore, the replacement of the matrix may not be an appropriate solution for improving cytoplasmic maturity. Changing the environment or adding growth factors alongside using combined hydrogels would be more efficient alternatives.

## Conclusion

As the results of this study indicated, CS was a permissive hydrogel. This biomaterial supports integrity of follicles, survival and development, including phenotypic maintenance, hormone production, normal gene expression, maturation and ovulation during 3D culture system.
